# Impact of prolonged requirement for insulin on 90-day mortality in critically ill patients without previous diabetic treatments: a post hoc analysis of the CONTROLING randomized control trial

**DOI:** 10.1186/s13054-022-04004-1

**Published:** 2022-05-16

**Authors:** François Thouy, Julien Bohé, Bertrand Souweine, Hassane Abidi, Jean-Pierre Quenot, Fabrice Thiollière, Jean Dellamonica, Jean-Charles Preiser, Jean-François Timsit, Vincent Brunot, Amna Klich, Nicholas Sedillot, Xavier Tchenio, Jean-Baptiste Roudaut, Nicolas Mottard, Hervé Hyvernat, Florent Wallet, Pierre-Eric Danin, Julio Badie, Richard Jospe, Jérôme Morel, Ali Mofredj, Abdelhamid Fatah, Jocelyne Drai, Anne Mialon, Ali Ait Hssain, Alexandre Lautrette, Eric Fontaine, Charles-Hervé Vacheron, Delphine Maucort-Boulch, Kada Klouche, Claire Dupuis

**Affiliations:** 1 Service de Médecine Intensive Réanimation, CHU Hôpital Gabriel-Montpied, 58 rue Montalembert, 63000 Clermont Ferrand, France; 2 Service d’Anesthésie-Réanimation-Médecine Intensive, Groupement hospitalier sud, Hospices Civils de Lyon, Pierre Bénite, France; 3 Service de Médecine Intensive Réanimation, CHU Dijon Bourgogne, Dijon, France; 4 Service de Médecine Intensive Réanimation, CHU Hôpital de L’Archet, Nice, France; 5 UR2CA Unité de Recherche Clinique Côte d’Azur, Université Côte d’Azur, Nice, France; 6 Department of Intensive Care, Erasme University Hospital, Université Libre de Bruxelles, Brussels, Belgium; 7 Service de Réanimation Médicale et des Maladies Infectieuses, Université Paris Diderot/Hôpital Bichat, Assistance Publique Hôpitaux de Paris, Paris, France; 8 Service de Médecine Intensive Réanimation, Centre Hospitalier Universitaire, Montpellier, France; 9 Service de Biostatistique - Bioinformatique, Pôle Santé Publique, Hospices Civils de Lyon, Lyon, France; 10 UMR5558, Laboratoire de Biométrie Et Biologie Évolutive, Équipe Biostatistique-Santé, CNRS, Villeurbanne, France; 11 Service de Réanimation, Hôpital Fleyriat, Bourg en Bresse, France; 12 Service de Réanimation Médico-Chirurgicale, CHU Hôpital de L’Archet, Nice, France; 13 Département d’Anesthésie et Réanimation, CHU, Saint Etienne, France; 14 Service de Réanimation, Hôpital du pays Salonais, Salon de Provence, France; 15 Service de Réanimation, Hôpital Pierre Oudot, Bourgoin Jallieu, France; 16 Laboratoire de Biochimie, Groupement Hospitalier Lyon Sud, Hospices Civils de Lyon, Lyon, France; 17 Département d’Anesthésie et Réanimation, Centre Jean Perrin, Clermont Ferrand, France; 18 INSERM U1055 - LBFA, University Grenoble Alpes, Grenoble, France

**Keywords:** Prolonged requirement for insulin, Critical care, Mortality

## Abstract

**Background:**

Stress hyperglycemia can persist during an intensive care unit (ICU) stay and result in prolonged requirement for insulin (PRI). The impact of PRI on ICU patient outcomes is not known. We evaluated the relationship between PRI and Day 90 mortality in ICU patients without previous diabetic treatments.

**Methods:**

This is a post hoc analysis of the CONTROLING trial, involving 12 French ICUs. Patients in the personalized glucose control arm with an ICU length of stay ≥ 5 days and who had never previously received diabetic treatments (oral drugs or insulin) were included. Personalized blood glucose targets were estimated on their preadmission usual glycemia as estimated by their glycated A1c hemoglobin (HbA1C). PRI was defined by insulin requirement. The relationship between PRI on Day 5 and 90-day mortality was assessed by Cox survival models with inverse probability of treatment weighting (IPTW). Glycemic control was defined as at least one blood glucose value below the blood glucose target value on Day 5.

**Results:**

A total of 476 patients were included, of whom 62.4% were male, with a median age of 66 (54–76) years. Median values for SAPS II and HbA1C were 50 (37.5–64) and 5.7 (5.4–6.1)%, respectively. PRI was observed in 364/476 (72.5%) patients on Day 5. 90-day mortality was 23.1% in the whole cohort, 25.3% in the PRI group and 16.1% in the non-PRI group (*p* < 0.01). IPTW analysis showed that PRI on Day 5 was not associated with Day 90 mortality (_IPTW_HR = 1.22; CI 95% 0.84–1.75; *p* = 0.29), whereas PRI without glycemic control was associated with an increased risk of death at Day 90 (_IPTW_HR = 3.34; CI 95% 1.26–8.83; *p* < 0.01).

**Conclusion:**

In ICU patients without previous diabetic treatments, only PRI without glycemic control on Day 5 was associated with an increased risk of death. Additional studies are required to determine the factors contributing to these results.

**Supplementary Information:**

The online version contains supplementary material available at 10.1186/s13054-022-04004-1.

## Background

Acute stress hyperglycemia results from an increase in hepatic gluconeogenesis and liver glucose output even when endogenous insulin levels are high (central insulin resistance) and a lower uptake of glucose by insulin-dependent glucose transporters (peripheral insulin resistance) [1]. It has been described to occur during the first hours after intensive care unit (ICU) admission and then to disappear over the first 12–48 h [2].

The hyperinsulinemic–euglycemic clamp is the standard procedure to define insulin resistance. It is useful for a better understanding of the pathophysiology of stress hyperglycemia at the early onset of the insult [3–6]. However, it cannot be easily performed in current practice and is more generally considered to be a laboratory research method. In non-diabetic patients, an alteration in glycemic homeostasis can be identified by a requirement for insulin to maintain the blood glucose level at its usual value. The usual blood glucose level, which is the blood glucose level prior to critical illness and ICU admission, is usually defined by diabetologists on the basis of the glycated hemoglobin A1c (HbA1C) level: UBGL = 28.7 × A1C − 46.7 (in mg/dl, with A1C in %) [7]. In the ICU, persistence in altered glycemic homeostasis several days after ICU admission can result in prolonged requirement for insulin (PRI). Although acute stress hyperglycemia is considered as an adaptive survival response [8], several studies have reported that insulin resistance is associated with severity of illness and poor outcome [3, 4, 9–13]. However, this poor outcome could be confounded with the occurrence of hypoglycemia due to the administration of exogenous insulin, which also increases the risk of death [14, 15]. All these studies were heterogeneous in terms of blood glucose target and population. None of them used the usual blood glucose target to drive exogenous insulin administration. The timing of administration of exogenous insulin has almost never been studied and reports on the effect of PRI on ICU patient outcome are scant [16]. In addition, depending on the timing of PRI occurrence, PRI could result from the severity of the initial insult leading to ICU admission or from subsequent ICU-acquired complications, such as a nosocomial infection, or from treatments such as steroids or parenteral nutrition.

CONTROLING (CONTROLe INdividualisé de la Glycémie) is a French multicentric randomized control trial (RCT) comparing maintenance of blood glucose level within a personalized blood glucose target range based on usual blood glucose level with maintenance of blood glucose level at 180 mg/dl or less [17–19] using the CPG (“Contrôle Personnalisé de la Glycémie,” or “personalized glycemic control”) algorithm (https://cpg.chu-lyon.fr) in all patients (Additional file 1).

The purpose of this post hoc analysis of the CONTROLING trial was to assess the impact on 90-day mortality of PRI on Day 5 after ICU admission in critically ill patients without previous diabetic treatments.

## Methods

### Study population

We performed a post hoc analysis of the CONTROLING RCT [19]. Briefly, in CONTROLING, patients were recruited from May 2015 to July 2016 from 12 ICUs taking part in the study. As part of routine care, all adult patients (> 18 years) admitted to the participating ICUs in whom spontaneous oral intake was not possible but who could receive enteral and/or parenteral nutrition and who were not expected to be discharged from the ICU within 2 days underwent blood sample measurement of their HbA1C level on ICU admission. In the intervention group, the blood glucose level was controlled to remain below the A1C derived usual blood glucose level + 15 mg/dL based on instructions from the CPG algorithm. In the CONTROLING trial, exclusion criteria were pregnancy, legal guardianship, previous enrollment in the study, admission to ICU for severe hypoglycemia, therapeutic limitation, patients with a medical history of diabetes who had received the transfusion of more than three red blood cell units over the 3 months prior to ICU admission and refusal to participate in the study.

In our study, we included all patients from the intervention group who had not previously received diabetic treatments (oral drugs or insulin) prior to ICU admission and who had an ICU length of stay of at least 5 days.

### Intervention

In the personalized glucose control group of the CONTROLING study, the HbA1C level at ICU admission was used to define the usual blood glucose level (UBGL) and the blood glucose target of each patient, where UBGL = 28.7 × HbA1C − 46.7 (in mg/dl, with HbA1C in %) [7]. The upper blood glucose target was calculated as UBGL + 15 mg/dl. For safety reasons, the upper limits of the blood glucose target were arbitrarily set at a minimum of 111 mg/dl (corresponding to an HbA1C level of 4.96%) and a maximum of 217 mg/dl (corresponding to an HbA1C level of 8.67%) (Additional file 1). Usual blood glucose level and blood glucose target values according to HbA1C levels are provided in Additional file 1: Table S1.

During the CONTROLING study, glycemia was controlled by the CPG web application, an electronic insulin infusion protocol (IIP), instructing the nurse to modulate glycemia, i.e., to schedule blood glucose assay, change the rate of insulin infusion and intravenously infuse dextrose for hypoglycemia. Regular insulin (50 IU in 50 mL of 0.9% sodium chloride) is continuously administered intravenously using an infusion pump.

### Other treatments

Except for blood glucose level management, patient care was left to the discretion of the attending physician (Additional file 1).

### Data collection

Data regarding the demographic and clinical characteristics of the patients were collected at baseline, including diabetes status based on medical history, the Charlson score, reason for ICU admission, the McCabe score and Simplified Acute Physiology Score II (SAPS II). Between randomization and ICU discharge, data concerning all blood glucose measurements, insulin administration, type and volume of all enteral and parenteral nutrition, body weight (measured daily), use of vasopressor support, non-prophylactic antimicrobial treatment, invasive mechanical ventilation (IMV) and renal replacement therapy (RRT) were collected. ICU length of stay (LOS) was recorded.

For 90-day mortality, participants or surrogates were contacted directly. All data were collected from the CPG web database, either during routine care or by the investigator for the purpose of the study.

### Definitions

PRI was defined as the need for exogenous insulin to maintain the blood glucose level under the upper limit of the personalized blood glucose target range based on usual blood glucose level on the fifth calendar day after ICU admission.

Glycemic control was defined simply as at least one glucose value under the blood glucose target value on the day of the assessment.

Three subgroups of patients were created according to insulin intake and achievement of glycemic control on Day 5: (1) no PRI, (2) PRI and glycemic control, and (3) PRI and no glycemic control.

Insulin intake was determined by the cumulative insulin doses administered to the patient per 24 h slots following ICU admission and expressed in units/day and in units/kg/day.

Calorie intake was determined by summing the calories received by the patient per 24 h, based on actual volume delivered, including dextrose calories and nutrition calories, and expressed in kcal/day and in kcal/kg/day. The insulin-to-calorie ratio per kilogram was also calculated and expressed in units/kcal/kg/day.

Adverse events were defined by the development of moderate and severe hypoglycemia. Moderate hypoglycemia was defined by a blood glucose level < 72 mg/dl (4 mmol/l) and severe hypoglycemia by a blood glucose level < 40 mg/dl (2.2 mmol/l).

### Objectives of the study

The first aim of our study was to assess the impact on Day 5 of PRI on 90-day mortality.

The second aim was to assess the impact on Day 5 of PRI on 90-day mortality according to whether glycemic control was achieved.

A subgroup analysis was carried out in non-diabetic patients defined by an HbA1C level under the 6.5% cutoff value to focus on true non-diabetic patients, since some diabetic patients without current antidiabetic treatments may not have been identified on ICU admission.

### Statistical analyses

Patient characteristics were expressed as *n* (%) for categorical variables and median [interquartile range (IQR)] for continuous variables. Comparisons were made with exact Fisher tests for categorical variables and Wilcoxon tests for continuous variables.

The primary outcome measure was 90-day mortality. We used an inverse probability of treatment weighting (IPTW) estimator, which is the inverse of the patients’ predicted probability of having PRI on Day 5 based on their baseline covariates. The IPTW estimator creates a pseudo-population in which baseline patient differences are balanced between treatment groups. The impact of PRI on Day 5 on 90-day mortality was estimated by a two-step process: (1) weight estimation by the IPTW estimator and (2) estimation of the impact of PRI on Day 5 on 90-day mortality using a weighted Cox model.

In a first step, the weight model, a non-parsimonious multivariable logistic regression model, was constructed to estimate each patient’s predicted probability of having PRI on Day 5.

All variables included in the weight model reflected knowledge available at baseline [20–22]. To avoid extreme weights, we used stabilized weights. The stabilized IPTWs were computed from the ratio of the mean probability of treatment in our cohort (numerator) to the estimated probabilities of treatment using baseline covariates (propensity score) (denominator). To ensure that the positivity assumption was obeyed, weights were truncated at the 1-99th percentile [23]. In a second step, we used a weighted Cox proportional hazard model to estimate the risk of death within the first 90 days of ICU stay after PRI on Day 5. A hazard ratio (HR) > 1 indicated an increased risk of death. The proportionality of hazard risk for having PRI on Day 5 was tested using Martingale residuals. A further analysis using a raw (non-weighted) multivariable Cox proportional hazard model was performed to confirm the results obtained with the IPTW. All models were stratified by center.

Similar analyses were performed for patients with an HbA1C level under 6.5%. Using similar IPTWs, we assessed the impact of PRI on Day 5 considering glycemic control.

For all tests, a two-sided *α* value of 0.05 was considered significant. There were no missing values. All statistical analyses were performed with SAS software, Version 9.4 (SAS Institute, Cary, NC).

## Results

### Main characteristics

**Fig. 1:**
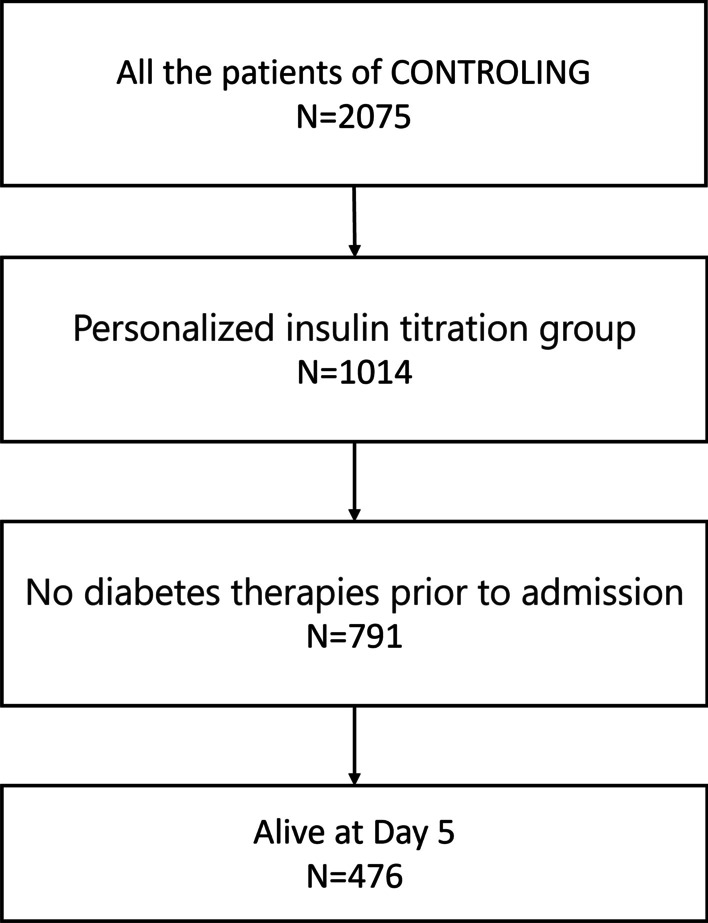
Flow chart

Of the 2075 patients enrolled in the CONTROLING RCT, 476 were included in our study (Fig. 1). Their main characteristics are reported in Table 1. They had a median age of 66 years [54–76] and 62.7% were male. Their median body mass index was 26.5 [23.5–30.7] kg/m^2^ and their median HbA1C level 5.7% [5.4–6.1]. A total of 413 (86.8%) patients were non-diabetic, with an HbA1C level ≤ 6.5%. Most of the patients had a medical reason for admission (77.9%). The median SAPS II score was 50 [37.5–64]. During their ICU stay, 79% required IMV, 24.2% RRT and 62.6% vasopressors.

**Table 1:** Baseline characteristics of the patients included at Day 5

Variables (*N* (%) or median [IQR])	All	No PRI	PRI	*P* value
Number of patients	476	112	364	
Age	66 [54; 76]	66.5 [53; 77]	66 [54; 76]	0.89
Gender (male)	297 (62.4)	67 (59.8)	230 (63.2)	0.52
BMI	26.5 [23.5; 30.7]	26.6 [23.9; 32.6]	26.4 [23.4; 30.4]	0.61
HbA1C (%)	5.7% [5.4–6.1]	5.9 [5.5; 6.3]	5.7 [5.3; 6.1]	< 0.01
HbA1C > 6.5%	77 (16.2)	24 (21.4)	53 (14.6)	0.08
Charlson score	2 [1; 3]	2 [1; 4]	2 [0; 3]	0.19
Medical motif of admission	371 (77.9)	90 (80.4)	281 (77.2)	0.36
SAPS II	50 [37.5; 64]	48.5 [36; 62.5]	50 [38; 64]	0.53
McCabe = Ultimately fatal	34 (7.1)	8 (7.1)	26 (7.1)	0.44
McCabe = Rapidly fatal	147 (30.9)	40 (35.7)	107 (29.4)	
McCabe = Non-fatal	295 (62)	64 (57.1)	231 (63.5)	
Delay before CPG start (hours)	23.8 [15.4; 47.7]	25 [15.9; 54.6]	23 [15.4; 46.9]	0.62
*At Day 5*				
Glycemic control at Day 5 (yes/no)	462 (97.1)	110 (98.2)	352 (96.7)	0.41
Cumulative insulin intake at day 5 (units)	65.1 [12.1; 152]	0 [0; 6.7]	97 [46.4; 183.6]	< 0.01
Calorie intakes (Kcal/kg/24H00)	15.3 [3.1; 20.8]	4.7 [1.4; 16.8]	16.9 [6.6; 21.4]	< 0.01
Insulin per calorie intakes (units/kcal) (× 1000)	23.9 [1.2; 48.9]	0 [0; 0]	33.9 [18.2; 62.5]	< 0.01
PRI (yes/no)	364 (76.5)		364 (100)	
No PRI	112 (23.5)	112 (100)	0 (0)	< 0.01
PRI and glycemic control	352 (73.9)	0 (0)	352 (96.7)	
PRI and no glycemic control	12 (2.5)	0 (0)	12 (3.3)	
Hypoglycemia (< 72 mmol/l)	48 (10.1)	2 (1.8)	46 (12.6)	< 0.01
Severe hypoglycemia (< 40 mmol/l)	0			
*Organ support in ICU*				
Mechanical ventilation (yes/no)	376 (79)	79 (70.5)	297 (81.6)	0.01
Noninvasive mechanical ventilation (yes/no)	292 (61.3)	57 (50.9)	235 (64.6)	< 0.01
Renal replacement therapy (yes/no)	115 (24.2)	25 (22.3)	90 (24.7)	0.60
Vasopressors (yes/no)	298 (62.6)	53 (47.3)	245 (67.3)	< 0.01
Number of days under mechanical ventilation	6 [2; 13]	3 [0; 9]	7 [3; 14]	< 0.01
Number of days under vasopressors	2 [0; 4]	0 [0; 2]	2 [0; 5]	< 0.01
ICU length of stay	10 [7; 17]	7 [5; 12]	11 [7; 18.5]	< 0.01
Death at day 90	110 (23.1)	18 (16.1)	92 (25.3)	0.04

*PRI* prolonged requirement for insulin, *SAPS* simplified acute physiology score, *BMI* body mass index, *CPG* “*contrôle personnalisé de la* glycémie,” or “personalized glycemic control”

On Day 5, median insulin intake was 24.3 units [1.1–51.9] and median calorie intake 1137.1 kcal [215.9–1631.6] or 15.3 kcal/kg [3.1–20.8]. Moderate hypoglycemia was recorded in 48 (10.1%) patients and none developed severe hypoglycemia. Median ICU LOS was 10 days [7–17] and 90-day mortality 23.1% (*N* = 110).

### Comparison of patients with and without PRI

Finally, 364 patients (76.5%) had PRI on Day 5. Comparisons of PRI and non-PRI patients are given in Table 1. PRI and non-PRI patients did not differ in age, Charlson score or SAPS II score (*p* values of 0.89, 0.19 and 0.53, respectively). PRI patients experienced more hypoglycemia, required more vasopressors and had higher calorie intake, longer duration of IMV, longer ICU LOS and a higher death rate at Day 90 (all *p* values < 0.01). Patients with PRI without glycemic control (*N* = 12) had HbA1C no higher than the others and did not present hypoglycemia on Day 5. However, the patients with PRI without glycemic control were more often under invasive mechanical ventilation and/or vasopressor and/or antimicrobial therapy on Day 4 (Additional file 1: Table S2).

### Construction of the propensity score

**Fig. 2:**
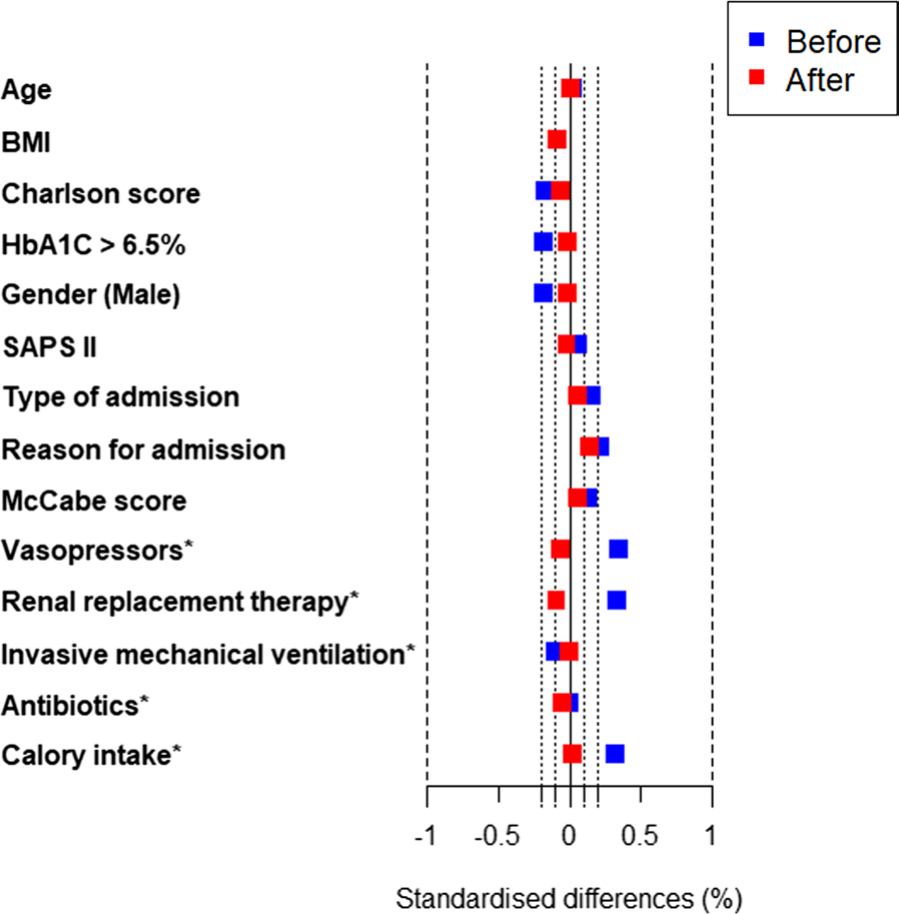
Standardized differences before and after weighting for IPTW. *On Day 4; *BMI* body mass index (kg/m^2^), *SAPS* simplified acute physiology score, *IPTW* inverse probability of treatment weight; calorie intake on day 4 (kcal/kg)

All the covariates were retained in the propensity score (Additional file 1: Table S3). After weighting, standardized differences were below 10%. Distribution of the propensity score in the subgroups of the patients with PRI and without PRI are given in Additional file 1: Figure S1. Standardized differences are reported before and after weighting for IPTW in Fig. 2.

### PRI on day 5 and 90-day mortality: results of the Cox models with weighting for IPTW

**Fig. 3:**
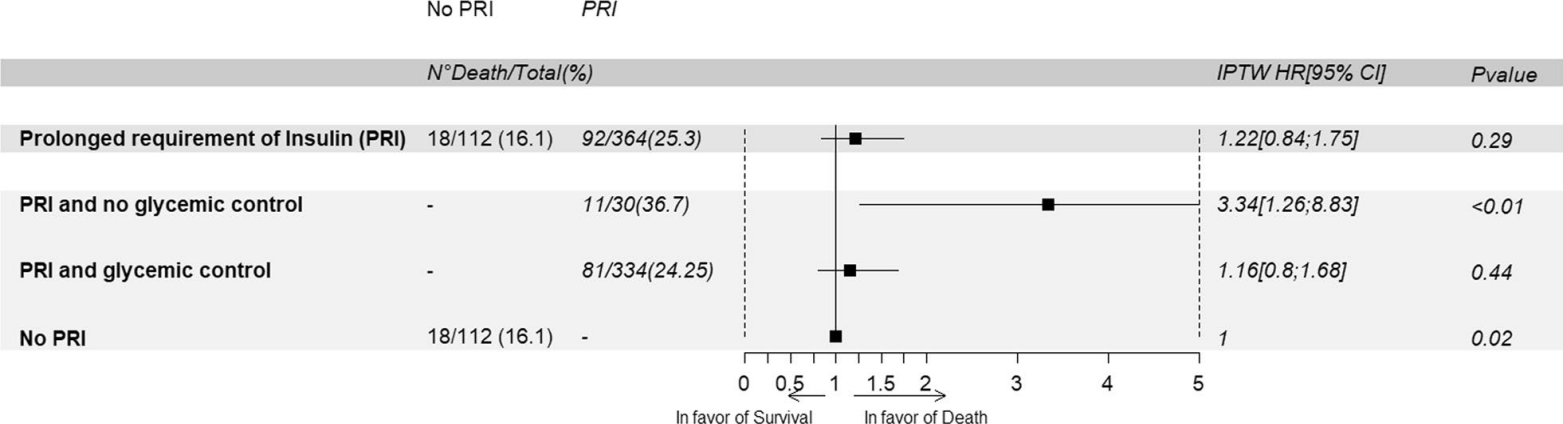
Association between insulin resistance and/or glycemic control with 90-day mortality: Cox model with weighting for IPTW. *PRI* prolonged requirement for insulin, *HR* hazard ratio, *CI* confidence interval, *IPTW* inverse probability of treatment weight

After weighting for IPTW, PRI on Day 5 was not associated with an increased risk of death (_IPTW_HR = 1.22; CI 95% 0.84–1.75; *p* value = 0.29) (Additional file 1: Table S4). In addition, PRI patients without glycemic control on Day 5 (*N* = 12) had a higher risk of death than patients with PRI and glycemic control (*N* = 352) (_IPTW_HR(PRI and no glycemic control/No PRI) = 3.34; CI 95% 1.26–8.83; *p* value < 0.01; _IPTW_HR(PRI and glycemic control/No PRI) = 1.16; CI 95% 0.8; 1.68; *p* value = 0.44) (Fig. 3, Additional file 1: Table S5). Similar results were obtained with the multivariate Cox model. In the subgroup of patients with an HbA1C under 6.5%, no patients had a PRI associated with an increased risk of death (Additional file 1: Tables S5–S6).

## Discussion

This study is one of the first to assess the effect of PRI on Day 5 in critically ill patients without previous diabetic treatments. We found that PRI on Day 5 was not associated with 90-day mortality, unless glycemia was not controlled. These results warrant several comments.

First, several strategies have been proposed to identify “altered glycemic homeostasis” The hyperinsulinemic–euglycemic clamp has been classically reported to assess insulin resistance in acute stress hyperglycemia, but cannot be easily performed in current practice [3–6, 24]. Other studies used a model-based measurement of insulin sensitivity to assess its evolution and its variability over time [24], which is, once again, difficult to perform in current practice. For this reason, several studies only assessed the prognostic effect of insulin administered to maintain blood glucose level below predefined thresholds [25, 26] with no distinction between diabetic and non-diabetic status [3, 6, 19, 24]. In our study, in patients without previous diabetic treatments, i.e., those for whom glycated hemoglobin measured at ICU admission could provide a reliable estimate of their usual glycemic levels, including undiagnosed diabetic patients (*n* = 63, with glycated hemoglobin > 6.5%), we used the requirement for insulin to target usual blood glucose level on Day 5 to define PRI. This is easy to do in current practice and physiologically appropriate.

In our study, the blood glucose target was consequently personalized and most often below the recommended blood glucose target level [27]. The median upper personalized blood glucose target was 131.3 mg/dL [122.7–142.8] (7.22 mmol/L [6.75–7.85]), which is below the recommended 180 mg/dL threshold that has been adopted in most intensive care units since NICE-SUGAR [28] and other recommendations [29]. In addition, we observed the usual blood glucose target levels between 140 and 180 mg/dL in patients with an HbA1C level between 6.5 and 7.3%, who accounted for only 7% of our total population (Additional file 1: Table S1). Therefore, concerning PRI, our results should not be confused with the association reported in many studies between hyperglycemia and risk of death in ICU patients [30]. Most often, these studies focused on glycemia at admission, included critically ill patients whether they had diabetes or not and used a single threshold for all patients to define hyperglycemia. Furthermore, the effect of insulin administration must also be taken into account to interpret our results and not only the effect of hyperglycemia.

Stress hyperglycemia has long been regarded as an adaptive and beneficial stress response, ensuring adequate cellular glucose uptake in non-insulin-dependent, obligatory glucose-consuming tissues such as the brain, phagocytes and reparative cells.

In our study, taking into account the severity of the patients and calorie intakes, PRI was not associated with an increased risk of death unless glycemic control was not obtained. Stress hyperglycemia seemed therefore not so deleterious if controllable by insulin [31].

Our results also underline an increased risk of mortality when glucose levels are not controlled. Similar results had already been reported [32, 33]. It has already been emphasized that patients harder to control with the highest glycemic levels and variability could have worse outcomes [14]. Very high hyperglycemia could be deleterious because of osmotic diuresis, predisposition to infectious complications and increased oxidative stress and inflammation [1, 33].

In that context, in cases of uncontrolled hyperglycemia, adaptation of glycemic and/or calorie intake should be discussed. Hypoglycemia could not explain the worse outcome of patients without glycemic control since none of them presented hypoglycemia on Day 5. However, these patients were more severely ill on Day 4. In that context, the impact of uncontrolled hyperglycemia could still be confounded with the critical illness even after weighting.

In our study, moderate hypoglycemia was more frequently observed in PRI patients than in the non-PRI subgroup, but none of the patients presented severe hypoglycemia and hypoglycemia on Day 5 was not associated with death. Usually, hypoglycemia is associated with worse outcomes in the ICU [34]. It generally occurs more often in patients with higher HbA1C [35] or high glycemic variations [36] and even more frequently in studies using tight glycemic controls [28]. In studies dealing with glycemic control, hypoglycemia is reported to be between 4.8 and 54% depending on the definitions of blood glucose target and hypoglycemia [37].

Finally, PRI seemed also to be related to calorie intake since PRI patients had a higher intake than those without PRI. After weighting, on calorie intakes, PRI was no longer associated with worse outcomes, finally questioning the benefit of lower calorie intake. Some data suggest that overfeeding could be harmful, especially during the period of hypercatabolism [38]. However, few studies have compared lower and higher doses of calorie intake in adult critically ill patients and most that have did not find any one strategy more beneficial than any other [39, 40].

### Limits and advantages

Our study has several strengths. First, we used a 90-day endpoint and weighted models. Second, we excluded those with previous diabetic treatments because of the difficulty in defining PRI in such patients. Third, our study is an ancillary study of the CONTROLING RCT, which is a guarantee of data quality.

Our study also has several limits. First, despite the use of propensity score analyses to draw causal inferences, the study was observational, and potential unmeasured confounders could still have biased our results. For this reason, we performed several sensitivity analyses.

Second, the choice of the usual blood glucose target based on HbA1C levels is open to criticism. Usual blood glucose targets are below the recommended 180 mg/dL target in critically ill patients and could be considered as tight blood glucose targets, which have been reported as harmful [28]. However, the aim of our study was not to compare different blood glucose targets but to assess, physiologically, the impact of PRI on Day 5 as defined by insulin administered to achieve a personalized blood glucose target within the patient’s current glycemia range prior to ICU admission.

Concerning the definition of uncontrolled hyperglycemia, we acknowledge that our definition of “glucose control” is unusual but it allows an easy dichotomous distribution between patients with and without glucose control. To the best of our knowledge, no specific definition of uncontrolled hyperglycemia has yet been reported. Consequently, our results concerning uncontrolled hyperglycemia based on this study definition should be interpreted with caution.

Third, we arbitrarily assessed PRI on Day 5. We could not accurately assess the impact of alteration of glycemic hemostasis before Day 5 since in the CONTROLING trial patients could be included up to 96 h after ICU admission. In addition, beyond Day 5, several time-dependent confounding factors related to ICU-acquired adverse events could develop, contributing to the persistence or development of PRI. We did not assess its cumulative effect over time and did not consider previous history of PRI in our patients in the analyses.

Fourth, the definition of PRI and the day of assessment were both defined after the CONTROLING RCT was carried out, but during the design of this observational study and before any analyses. Fifth, the results cannot be extrapolated to diabetic patients, for whom PRI is even harder to define. Finally, the heterogeneity of the method of glycemic measurement could have affected the results. Also, we did not consider renal clearance, which could influence blood insulin concentration and therefore PRI.

## Conclusion

In critically ill patients without previous diabetic treatments, using a personalized target for glycemic control based on individual usual glycemia, mortality at Day 90 was higher for patients with uncontrolled hyperglycemia at Day 5. On the other hand, in patients with controlled hyperglycemia, mortality at Day 90 was not modified by the administration of insulin on Day 5. Further studies should focus on the subgroup of patients with uncontrolled hyperglycemia and seek the factors contributing to these results, including persistent acute illness, emerging critical care complications, need for adaptive stress hyperglycemia and excess calorie intake.

## Supplementary Information

**Additional file 1**. **Table S1:** Glycemic targets according to HbA1c level and distribution of patients according to HbA1c. **Table S2:** Characteristics of the patients included at Day 5 depending on PRI and glycemic control status. **Table S3:** Propensity score: having PRI on day 5. Multivariate logistic regression model. **Figure S1:** Histogram of the distribution of the propensity score (having Prolonged requirement of insulin at day 5). **Table S4:** Main results with weight truncation as sensitivity analyses – impact of PRI on the occurrence of death before day 90 among, a cox survival model with ponderation on IPTW. **Table S5:** Sensitivity analyses: multivariate Cox hazard model without weighting for IPTW: association between PRI and the occurrence of death before day 90. **Table S6:** Subgroup analysis of patients with an HbA1C <=6.5%: association between PRI and/or glycemic control and 90-day mortality.

## Data Availability

Data are available on request to the corresponding author.

## Abbreviations

CONTROLING: CONTROLe INdividualisé de la Glycémie
CPG: Contrôle Personnalisé de la Glycémie, or “personalized glycemic control”
HbA1C: Glycated A1c hemoglobin
HR: Hazard ratio
ICU: Intensive care unit
IMV: Invasive mechanical ventilation
IPTW: Inverse probability of treatment weight
IQR: Interquartile range
LOS: Length of stay
PRI: Prolonged requirement for insulin
RCT: Randomized control trial
RRT: Renal replacement therapy
SAPS: Simplified acute physiology score
UBGL: Usual blood glucose level

## References

[1] Van Cromphaut SJ (2009). Hyperglycaemia as part of the stress response: the underlying mechanisms. Best Pract Res Clin Anaesthesiol.

[2] Thomas F, Pretty CG, Fisk L, Shaw GM, Chase JG, Desaive T (2014). Reducing the impact of insulin sensitivity variability on glycaemic outcomes using separate stochastic models within the STAR glycaemic protocol. Biomed Eng Online.

[3] Mukherjee K, Sowards KJ, Brooks SE, Norris PR, Jenkins JM, Smith MA (2015). Insulin resistance in critically injured adults: contribution of pneumonia, diabetes, nutrition, and acuity. Surg Infect.

[4] Donatelli F, Nafi M, Di Nicola M, Macchitelli V, Mirabile C, Lorini L (2015). Twenty-four hour hyperinsulinemic-euglycemic clamp improves postoperative nitrogen balance only in low insulin sensitivity patients following cardiac surgery. Acta Anaesthesiol Scand.

[5] DeFronzo RA, Tobin JD, Andres R (1979). Glucose clamp technique: a method for quantifying insulin secretion and resistance. Am J Physiol.

[6] Mukherjee K, Sowards KJ, Brooks SE, Norris PR, Boord JB, May AK (2014). Insulin resistance increases before ventilator-associated pneumonia in euglycemic trauma patients. Surg Infect.

[7] Nathan DM, Kuenen J, Borg R, Zheng H, Schoenfeld D, Heine RJ (2008). Translating the A1C assay into estimated average glucose values. Diabetes Care.

[8] Marik PE, Bellomo R (2013). Stress hyperglycemia: an essential survival response!. Crit Care.

[9] Zauner A, Nimmerrichter P, Anderwald C, Bischof M, Schiefermeier M, Ratheiser K (2007). Severity of insulin resistance in critically ill medical patients. Metabolism.

[10] Pieracci F, Hydo L, Eachempati S, Pomp A, Shou J, Barie PS (2008). Higher body mass index predicts need for insulin but not hyperglycemia, nosocomial infection, or death in critically ill surgical patients. Surg Infect.

[11] Casaer MP, Hermans G, Wilmer A, Van den Berghe G (2011). Impact of early parenteral nutrition completing enteral nutrition in adult critically ill patients (EPaNIC trial): a study protocol and statistical analysis plan for a randomized controlled trial. Trials.

[12] Yan C-L, Huang Y-B, Chen C-Y, Huang G-S, Yeh M-K, Liaw W-J (2013). Hyperglycemia is associated with poor outcomes in surgical critically ill patients receiving parenteral nutrition. Acta Anaesthesiol Taiwanica Off J Taiwan Soc Anesthesiol.

[13] De Vlieger G, Ingels C, Wouters PJ, Debaveye Y, Vanhorebeek I, Wauters J (2019). Impact of supplemental parenteral nutrition early during critical illness on invasive fungal infections: a secondary analysis of the EPaNIC randomized controlled trial. Clin Microbiol Infect Off Publ Eur Soc Clin Microbiol Infect Dis.

[14] Uyttendaele V, Dickson JL, Shaw GM, Desaive T, Chase JG (2017). Untangling glycaemia and mortality in critical care. Crit Care Lond Engl.

[15] Falciglia M, Freyberg RW, Almenoff PL, D’Alessio DA, Render ML (2009). Hyperglycemia-related mortality in critically ill patients varies with admission diagnosis. Crit Care Med.

[16] Marik PE, Bellomo R (2013). Stress hyperglycemia: an essential survival response!. Crit Care Med.

[17] Handelsman Y, Bloomgarden ZT, Grunberger G, Umpierrez G, Zimmerman RS, Bailey TS (2015). American association of clinical endocrinologists and american college of endocrinology—clinical practice guidelines for developing a diabetes mellitus comprehensive care plan—2015. Endocr Pract Off J Am Coll Endocrinol Am Assoc Clin Endocrinol.

[18] Ichai C, Preiser J-C, Société Française d’Anesthésie-Réanimation, Société de Réanimation de langue Française, Experts group (2010). International recommendations for glucose control in adult non diabetic critically ill patients. Crit Care Lond Engl.

[19] Bohé J, Abidi H, Brunot V, Klich A, Klouche K, Sedillot N (2021). Individualised versus conventional glucose control in critically-ill patients: the CONTROLING study-a randomized clinical trial. Intensive Care Med.

[20] Austin PC, Stuart EA (2015). Moving towards best practice when using inverse probability of treatment weighting (IPTW) using the propensity score to estimate causal treatment effects in observational studies. Stat Med.

[21] Moore KL, Neugebauer R, Laan MJ, Tager IB (2012). Causal inference in epidemiological studies with strong confounding. Stat Med.

[22] Austin PC, Grootendorst P, Anderson GM (2007). A comparison of the ability of different propensity score models to balance measured variables between treated and untreated subjects: a Monte Carlo study. Stat Med.

[23] Hernán MA, Robins JM. Causal inference (2016).

[24] Sah Pri A, Chase JG, Pretty CG, Shaw GM, Preiser J-C, Vincent J-L (2014). Evolution of insulin sensitivity and its variability in out-of-hospital cardiac arrest (OHCA) patients treated with hypothermia. Crit Care Lond Engl.

[25] De La Rosa G, Vasquez EM, Quintero AM, Donado JH, Bedoya M, Restrepo AH (2013). The potential impact of admission insulin levels on patient outcome in the intensive care unit. J Trauma Acute Care Surg.

[26] Basi S, Pupim LB, Simmons EM, Sezer MT, Shyr Y, Freedman S (2005). Insulin resistance in critically ill patients with acute renal failure. Am J Physiol-Ren Physiol.

[27] Bohé J, Preiser J-C (2022). Individualized glycaemic management for critically ill patients. Authors’ reply. Intensive Care Med.

[28] Finfer S, Chittock DR, Su SYS, Blair D, Foster D, NICE-SUGAR Study Investigators (2009). Intensive versus conventional glucose control in critically ill patients. N Engl J Med.

[29] Rhodes A, Evans LE, Alhazzani W, Levy MM, Antonelli M, Ferrer R (2017). Surviving sepsis campaign: international guidelines for management of sepsis and septic shock: 2016. Intensive Care Med.

[30] Whitcomb BW, Pradhan EK, Pittas AG, Roghmann M-C, Perencevich EN (2005). Impact of admission hyperglycemia on hospital mortality in various intensive care unit populations. Crit Care Med.

[31] Koyfman L, Brotfain E, Frank D, Bichovsky Y, Kovalenko I, Benjamin Y (2018). The clinical significance of hyperglycemia in nondiabetic critically ill multiple trauma patients. Ther Adv Endocrinol Metab.

[32] der Voort PHJ, Feenstra RA, Bakker AJ, Heide L, Boerma EC, der Horst ICC (2006). Intravenous glucose intake independently related to intensive care unit and hospital mortality: an argument for glucose toxicity in critically ill patients. Clin Endocrinol (Oxf).

[33] Losser M-R, Damoisel C, Payen D (2010). Bench-to-bedside review: glucose and stress conditions in the intensive care unit. Crit Care.

[34] Egi M, Bellomo R, Stachowski E, French CJ, Hart GK, Taori G (2010). Hypoglycemia and outcome in critically ill patients. Mayo Clin Proc.

[35] Egi M, Krinsley JS, Maurer P, Amin DN, Kanazawa T, Ghandi S (2016). Pre-morbid glycemic control modifies the interaction between acute hypoglycemia and mortality. Intensive Care Med.

[36] Donati A, Damiani E, Domizi R, Botticelli L, Castagnani R, Gabbanelli V (2014). Glycaemic variability, infections and mortality in a medical-surgical intensive care unit. Crit Care Resusc J Australas Acad Crit Care Med.

[37] Braithwaite SS, Bavda DB, Idrees T, Qureshi F, Soetan OT (2017). Hypoglycemia reduction strategies in the ICU. Curr Diab Rep.

[38] Moonen HPFX, Beckers KJH, van Zanten ARH (2021). Energy expenditure and indirect calorimetry in critical illness and convalescence: current evidence and practical considerations. J Intensive Care.

[39] Al-Dorzi HM, Albarrak A, Ferwana M, Murad MH, Arabi YM (2016). Lower versus higher dose of enteral caloric intake in adult critically ill patients: a systematic review and meta-analysis. Crit Care Lond Engl.

[40] Arabi YM, Aldawood AS, Haddad SH, Al-Dorzi HM, Tamim HM, Jones G (2015). Permissive underfeeding or standard enteral feeding in critically ill adults. N Engl J Med.

