# Supplementary material for: Impact of prolonged requirement for insulin on 90-day mortality in critically ill patients without previous diabetic treatments: a post hoc analysis of the CONTROLING randomized control trial
Source: Crit Care. 2022 May 16;26:138. doi: 10.1186/s13054-022-04004-1 (PMC9109308; doi:10.1186/s13054-022-04004-1)
Supplement: Supplementary file 1 — Additional file 1. Table S1: Glycemic targets according to HbA1c level and distribution of patients according to HbA1c. Table S2: Characteristics of the patients included at Day 5 depending on PRI and glycemic control status. Table S3: Propensity score: having PRI on day 5. Multivariate logistic regression model. Figure S1: Histogram of the distribution of the propensity score (having Prolonged requirement of insulin at day 5). Table S4: Main results with weight truncation as sensitivity analyses – impact of PRI on the occurrence of death before day 90 among, a cox survival model with ponderation on IPTW. Table S5: Sensitivity analyses: multivariate Cox hazard model without weighting for IPTW: association between PRI and the occurrence of death before day 90. Table S6: Subgroup analysis of patients with an HbA1C <=6.5%: association between PRI and/or glycemic control and 90-day mortality. [file 13054_2022_4004_MOESM1_ESM.docx]

# Additional file 1

## Impact of prolonged requirement for insulin on 90-day mortality in critically ill patients without previous diabetic treatments– a post hoc analysis of the CONTROLING randomized control trial

## The CPG web application to control blood glucose level.

An electronic insulin infusion protocol (IIP) was first developed in 2005 by two of the authors (JB and HA) in the university hospital of Lyon, France. It was named CSG (*Contrôle Strict de la Glycémie*) for “strict glycemic control” with a blood glucose target set at 80-105 mg/dL (4.5-5.9 mmol/L). In 2009 the protocol was renamed CPG (*Contrôle Personnalisé de la Glycémie*) for “personalized glycemic control” in which the personalized blood glucose target (in mg/dl) was calculated on the basis of the HbA1C level (in %) by the formula 28.7 × HbA1C – 61.1 to 28.7 × HbA1C – 32.3. To avoid maintaining blood glucose at a too low or too high level, the minimum and maximum limits for the personalized target ranges were arbitrarily set at 81 mg/dl (corresponding to an HbA1C level of 4.96 %) and 217 mg/dl (corresponding to an HbA1C level of 8.67 %), respectively. Whenever the HbA1C level is not reported in the CPG application, the blood glucose target is set from 151 mg/dl to 180 mg/dl. The IIP was first used as a program installed on bedside computers. From September 2012, it has been freely accessible on the internet (https://cpg.chu-lyon.fr). Since its first applications, the IIP has become part of standard care for most patients admitted to the ICU. The CPG web application is currently used in 12 ICUs in France. Since September 2012, nearly 29,000 patients have had their blood glucose level checked by this application.

The IIP is based on multiple insulin infusion rate sliding scales and rules to move within a scale and from one scale to another.

Depending on the level and stability of the previous blood glucose measurements, glycemic checks are performed from 1 to 6 hours apart.

Whenever blood glucose determination is below 63 mg/dl (3.5 mmol/L), the CPG web application indicates that insulin infusion should be discontinued, and 20 to 30 mL 30% dextrose be immediately administered. The next blood glucose control is scheduled one hour later. Whenever blood glucose determination is below 45 mg/dl (2.5 mmol/L), the CPG web application asks nurses to send a blood sample to the laboratory to check the levels of glucose (blood sample taken from a fluoride tube) and potassium (to correct possible associated hypokalemia). Hypoglycemia is corrected without waiting for the results of blood glucose verification performed in the laboratory.

When required by the IIP, regular insulin (50 IU in 50 mL of 0.9 percent sodium chloride) is continuously administered intravenously with the use of an infusion pump.

The IIP use does not rely on a physician’s input or interpretation. Nurse-work is restricted to following program instructions strictly, *ie* to schedule the determination of blood glucose, change the rate of insulin infusion, and intravenously infuse dextrose in case of hypoglycemia.

## HbA1C assessment and point-of-care glucose meters

**HbA1C assessment:**

Hemoglobin HbA1c assays were performed in 10 laboratories of which 2 (Lyon and Nice) carried out measurements for two centers.

Seven laboratories conducted the assay, using either high performance liquid chromatography on Variant II Biorad © (Nice, Lyon, Trévenans, Saint Etienne, Clermont) or Tosoh G8© (Dijon and Montpellier).

Three laboratories performed the assay using automated immunometric techniques, two by capillary electrophoresis on SEBIA© Capillaris automate or minicap (Bourgoin-Jallieu and Salon de Provence) and one by turbidimetric immunoinhibition dosage (Bourg en Bresse) on DCX Beckman Coulter©.

All assay techniques were calibrated according to the International Federation Clinical Chemistry (IFCC) requirements for HbA1c testing. The results were reported as % HbA1c and mmol HbA1c / mmol hemoglobin. The HPLC Biorad© and Tosoh© chromatography techniques and the capillary electrophoresis technique yield the same results and eliminate interferences related to hemoglobin variants or high fetal hemoglobin levels. The turbidimetric immunoinhibition technique does not detect the presence of hemoglobin variants and, in this event, will lower the results.

**Point-of-care glucose meters**

The following point-of-care glucose meters were used during the time course of the study:

- Accu-Chek Performa, Roche Diabetes Care (Switzerland) in Montpellier and Bourgoin Jallieu;
- Contour XT, Ascensia Diabetes Care (Switzerland) in Dijon;
- FreeStyle Optium Néo, Abbott Diabetes Care Ltd (UK) in Clermont-Ferrand and Salon de Provence;
- FreeStyle Optium Xceed, Abbott Diabetes Care Ltd (UK) in Lyon;
- StatTrip Xpress, Nova Biomedical (USA) in Bourg en Bresse, Nice, Saint Etienne, and Trévenans.

## Nutrition

Each unit complied with the latest nutritional recommendations of French learned societies^[[1]](#footnote-1)^. A central point of these recommendations was the early initiation of enteral nutrition with a calorie target of 20-25 kCal/kg/d. If enteral nutrition was contraindicated, parenteral nutrition was initiated on the third day after ICU admission. Enteral or parenteral nutrition was administered under continuous flow.

For 6 out of 12 centers, nutritional management was driven by CPG and followed the latest recommendations of French learned societies. In CPG, the in-charge physician documented the possible contraindication to enteral or parenteral nutrition in order to allow the application to provide prescriptions to the nurse for enteral and parenteral nutrition, vitamins, trace elements, and antiemetic drugs if vomiting occurred.

Tables and Figures

Table S 1: Glycemic targets according to HbA1c level and distribution of patients according to HbA1c

|  | TARGETS | | N (%) on Day 5 | |
| --- | --- | --- | --- | --- |
| HbA1c |  |  | Patients | PRI |
|  | LOW | HIGH |  |  |
| ≤ 5% | - | 114 mg/dl | 51 (10.7) | 46 (90.2) |
| >5 et ≤ 5.5% | 82 mg/dl | 129 mg/dl | 132 (27.7) | 106 (80.3) |
| >5.5 and ≤ 6% | 96 mg/dl | 144 mg/dl | 150 (31.5) | 113 (75.3) |
| >6 and ≤6.5% | 111 mg/dl | 158 mg/dl | 80 (16.8) | 57 (71.25) |
| >6.5 and ≤7% | 125 mg/dl | 172 mg/dl | 32 (6.7) | 22 (68.8) |
| >7 and ≤7.5% | 140 mg/dl | 187 mg/dl | 16 (3.4) | 9 (56.25) |
| >7.5 and ≤8% | 154 mg/dl | 201 mg/dl | 6 (1.3) | 5 (83.3) |
| >8 and ≤8.5% | 169 mg/dl | 215 mg/dl | 3 (0.6) | 1 (33.3) |
| >8.5 | 183 mg/dl | 220 mg/dl | 6 (1.3) | 5 (83.3) |

Table S 2: Characteristics of the patients included at Day 5 depending on PRI and glycemic control status

| Variables  (median[IQR]/N(%)) | No PRI | PRI and  glycemic control | PRI and  no glycemic control | . |
| --- | --- | --- | --- | --- |
| Number of patients | 112 (100) | 352 (100) | 12 (100) | . |
| Age | 66.5 [53 ; 77] | 66 [54.5 ; 76] | 58.5 [53.5 ; 66.5] | 0.35 |
| HbA1C | 5.9 [5.5 ; 6.3] | 5.7 [5.3 ; 6.1] | 5.55 [5.25 ; 6.45] | <0.01 |
| HbA1C> 6.5% | 24 (21.43) | 50 (14.2) | 3 (25) | 0.14 |
| Charlson score | 2 [1 ; 4] | 2 [0 ; 3] | 2 [1 ; 3] | 0.35 |
| SAPS II | 48.5 [36 ; 62.5] | 50 [38 ; 64] | 52 [36.5 ; 65.5] | 0.82 |
| Maximal glycemia at Day 5 | 7.21 [6.34 ; 8.11] | 8.95 [7.9 ; 10.75] | 9.57 [7.81 ; 12.28] | <0.01 |
| Minimal glycemia at Day 5 | 5.65 [4.94 ; 6.09] | 5.32 [4.47 ; 6.07] | 8.22 [6.98 ; 9.1] | <0.01 |
| Hypoglycemia | 2 (1.79) | 46 (13.07) | 0 | <0.01 |
| Variables the day before |  |  |  |  |
| Calory intake (Kcal/kg/24H00) | 6.09 [1.43 ; 15.89] | 15.31 [4.23 ; 20.59] | 2.59 [1.12 ; 15.32] | <0.01 |
| Vasopressors | 16 (14.29) | 97 (27.56) | 7 (58.33) | <0.01 |
| Invasive mechanical ventilation | 75 (66.96) | 285 (80.97) | 12 (100) | <0.01 |
| Renal replacement therapy | 14 (12.5) | 34 (9.66) | 1 (8.33) | 0.67 |
| antimicrobial therapy | 89 (79.46) | 279 (79.26) | 11 (91.67) | 0.57 |
| 90 day mortality | 18 (16.07) | 85 (24.15) | 7 (58.33) | <0.01 |

PRI: Prolonged requirement for Insulin; SAPS: Simplified Acute Physiology Score

*Table S 3: Propensity score: having PRI on day 5. Multivariate logistic regression model*

|  | OR |  | IC 95% | |  |  | p-value |
| --- | --- | --- | --- | --- | --- | --- | --- |
| BMI > 30 kg/m² | 1.03 | [ | 0.31 | ; | 3.46 | ] | 0.96 |
| 25 and 30 kg/m² | 1.07 | [ | 0.33 | ; | 3.52 | ] | 0.91 |
| 18 and 25 kg/m² | 0.91 | [ | 0.28 | ; | 2.94 | ] | 0.87 |
| ≤ 18 kg/m² | 1 |  |  |  |  |  | 0.95 |
| HbA1C > 6.5% | 0.65 | [ | 0.36 | ; | 1.18 | ] | 0.16 |
| Sex (Male) | 0.86 | [ | 0.54 | ; | 1.39 | ] | 0.55 |
| Charlson score > 2 | 0.77 | [ | 0.36 | ; | 1.62 | ] | 0.49 |
| 1-2 | 0.62 | [ | 0.32 | ; | 1.2 | ] | 0.15 |
| = 0 | 1 |  |  |  |  |  | 0.33 |
| Type of admission Urgent surgery | 1.6 |  | 0.72 | ; | 3.53 | ] | 0.25 |
| Elective surgery | 0.61 |  | 0.27 | ; | 1.41 | ] | 0.25 |
| Medical | 1 |  |  |  |  |  | 0.21 |
| Reason for admission Neurological | 1.07 | [ | 0.47 | ; | 2.44 | ] | 0.88 |
| Others | 0.87 | [ | 0.39 | ; | 1.97 | ] | 0.74 |
| Respiratory | 1.6 | [ | 0.76 | ; | 3.38 | ] | 0.22 |
| Digestive | 1.09 | [ | 0.44 | ; | 2.7 | ] | 0.86 |
| Cardiovascular | 1 |  |  |  |  |  | 0.46 |
| SAPS II | 1 | [ | 0.99 | ; | 1.01 | ] | 0.89 |
| McCabe score Non-fatal | 1.12 | [ | 0.43 | ; | 2.9 | ] | 0.82 |
| Rapidly fatal | 1.4 | [ | 0.52 | ; | 3.76 | ] | 0.5 |
| Ultimately fatal | 1 |  |  |  |  |  | 0.67 |
| Renal replacement therapy on Day 4 | 0.62 | [ | 0.29 | ; | 1.34 | ] | 0.23 |
| Antibiotics on Day 4 | 0.71 | [ | 0.39 | ; | 1.28 | ] | 0.25 |
| Vasopressors on Day 4 | 2.48 | [ | 1.29 | ; | 4.78 | ] | 0.01 |
| Invasive mechanical ventilation on Day 4 | 1.44 | [ | 0.83 | ; | 2.51 | ] | 0.19 |
| Calorie intake > 20 kcal/kg | 3.45 | [ | 1.74 | ; | 6.82 | ] | <0.01 |
| on Day 4 between 15 and 20 kcal/kg | 2.71 | [ | 1.34 | ; | 5.48 | ] | 0.01 |
| between 10 and 15 kcal/kg | 1.42 | [ | 0.81 | ; | 2.51 | ] | 0.23 |
| < 10 kcal/kg | 1 |  |  |  |  |  | <0.01 |

c-index 0.75; Hosmer and Lemshow test: 0.33; PRI: prolonged requirement for insulin; BMI: Body Mass Index; SAPS II: Simplified Acute Physiology Score


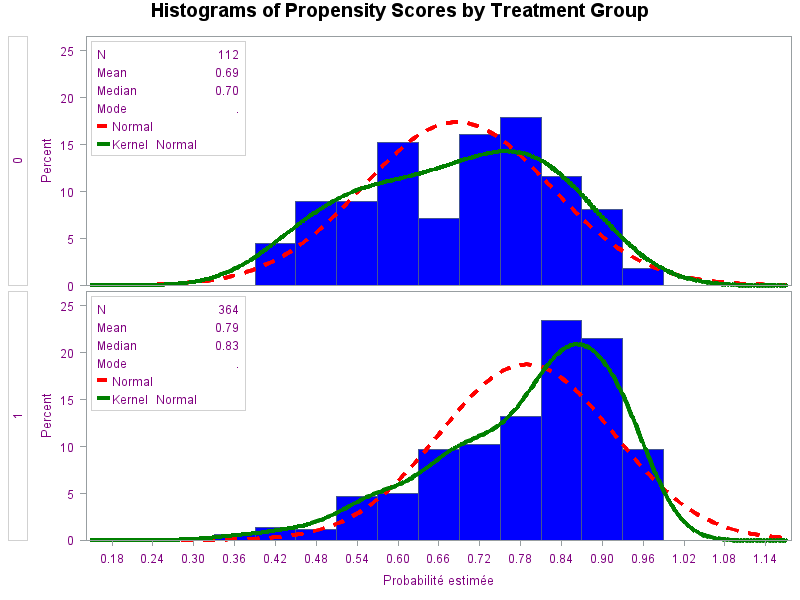


Probability distribution

Figure S 1: Histogram of the distribution of the propensity score (having Prolonged requirement of insulin at day 5)

Table S 4: Main results with weight truncation as sensitivity analyses – impact of PRI on the occurrence of death before day 90 among, a cox survival model with ponderation on IPTW

| Weight with truncation | Mean(Sd) | Min/max | _IPTW_HR | CI 95% | P value |
| --- | --- | --- | --- | --- | --- |
| Day 5 |  |  |  |  |  |
| 0/100 | 1/0.914 | 0.519/9.459 | 1.22 | [0.85 ; 1.76] | 0.28 |
| 1/99 | 0.997/0.896 | 0.524/8.542 | 1.22 | [0.84 ; 1.75] | 0.29 |
| 5/95 | 0.971/0.76 | 0.533/4.467 | 1.18 | [0.82 ; 1.7] | 0.38 |
| 10/90 | 0.953/0.694 | 0.544/3.651 | 1.22 | [0.84 ; 1.77] | 0.30 |
| 25/75 | 0.907/0.559 | 0.569/2.49 | 1.37 | [0.92 ; 2.03] | 0.12 |
| 50/50 | 0.863/0.452 | 0.612/1.678 | 1.72 | [1.11 ; 2.65] | 0.01 |

Sd: standard deviation, HR: Hazard Ratio, CI: Confidence Intervals, IPTW: Inverse Probability of Treatment Weight

Table S 5: Sensitivity analyses: multivariate Cox hazard model without weighting for IPTW: association between PRI and the occurrence of death before day 90.

| Variable | HR | CI 95% | Pvalue |
| --- | --- | --- | --- |
| Prolonged Requirement for Insulin (PRI) | 1.62 | [0.99 ; 2.64] | 0.05 |
| PRI and no glycemic control | 3.10 | [1.48 ; 6.49] | <0.01 |
| PRI and glycemic control | 1.50 | [0.91 ; 2.46] | 0.11 |
| No PRI | 1 |  | 0.01 |

Multivariable model adjusted for the following covariates: SAPS II, Type of ICU stay, main reason for admission, McCabe score, HbA1c.

PRI: Prolonged Requirement for Insulin; HR: Hazard Ratio; CI: Confidence Intervals; IPTW: Inverse of probability of treatment weight

Table S 6: Subgroup analysis of patients with an HbA1C <=6.5%: association between PRI and/or glycemic control and 90-day mortality

|  | _IPTW_HR | CI 95% | pvalue |
| --- | --- | --- | --- |
| Prolonged Requirement for Insulin (PRI) | 1.23 | [0.83 ; 1.84] | 0.30 |
| PRI and no glycemic control | 2.41 | [0.82 ; 7.05] | 0.11 |
| PRI and glycemic control | 1.21 | [0.81 ; 1.8] | 0.360 |
| No PRI | 1 |  | 0.24 |

PRI: Prolonged Requirement for Insulin; HR: Hazard Ratio; CI: Confidence Intervals; IPTW: Inverse of probability of treatment weight

1. [↑](#footnote-ref-1)
